# Comparison of Hepatotoxicity Associated With New BCR-ABL Tyrosine Kinase Inhibitors vs Imatinib Among Patients With Chronic Myeloid Leukemia: A Systematic Review and Meta-analysis

**DOI:** 10.1001/jamanetworkopen.2021.20165

**Published:** 2021-07-22

**Authors:** Zhe Wang, Xiaoyu Wang, Zhen Wang, Yuyi Feng, Yaqin Jia, Lili Jiang, Yangliu Xia, Jun Cao, Yong Liu

**Affiliations:** 1 School of Life and Pharmaceutical Sciences, Dalian University of Technology, Panjin, China; 2 Department of Occupational and Environmental Health, Dalian Medical University, Dalian, China

## Abstract

**Importance:**

Although BCR-ABL fusion oncoprotein tyrosine kinase inhibitors (BCR-ABL TKIs) can substantially improve the survival rate of chronic myeloid leukemia (CML), they are clinically accompanied by severe hepatotoxicity.

**Objective:**

To compare the relative risk (RR) of hepatotoxicity of new-generation BCR-ABL TKIs with that of imatinib, and to provide an overall assessment of the clinical benefit.

**Data Sources:**

PubMed, Embase, Cochrane library databases, and ClinicalTrials.gov were searched for clinical trials published between January 2000 and April 2020.

**Study Selection:**

Study selection was conducted independently by 2 investigators according to the inclusion and exclusion criteria published previously in the protocol: only randomized phase 2 or phase 3 clinical trials that compared bosutinib, dasatinib, nilotinib, or ponatinib with imatinib were included. Among the 2666 records identified, 9 studies finally fulfilled the established criteria.

**Data Extraction and Synthesis:**

Two investigators extracted study characteristics and data independently using a standardized data extraction form. Data were extracted according to Preferred Reporting Items for Systematic Reviews and Meta-analyses (PRISMA) reporting guidelines. When substantial heterogeneity was observed, pooled estimates were calculated based on the random-effect model; otherwise, the fixed-effect model was used.

**Main Outcomes and Measures:**

Data extracted included study characteristics, baseline patient information, interventions and data on all-grade and high-grade (grades 3 and 4) elevation of alanine aminotransferase (ALT) and aspartate aminotransferase (AST) levels, overall survival, and major molecular response (MMR). The RRs and 95% CIs were calculated using the inverse variance method.

**Results:**

Nine trials involving 3475 patients were analyzed; the median (range) age was 49 (18-91) years; 2059 (59.2%) were male patients. Increased risks were observed for each new-generation TKI except for dasatinib. Patients receiving new-generation TKIs were more likely to experience all grades of ALT elevation (pooled RR, 2.89; 95% CI, 1.78-4.69; *P* < .001) and grades 3 and 4 ALT elevation (pooled RR, 4.36; 95% CI, 2.00-9.50; *P* < .001) compared with those receiving imatinib. Patients receiving new-generation TKIs were also more likely to experience all grades of AST elevation (pooled RR, 2.20; 95% CI, 1.63-2.98; *P* < .001) and grades 3 and 4 AST elevation (pooled RR, 2.65; 95% CI, 1.59-4.42; *P* < .001) compared with those receiving imatinib. New-generation TKIs were associated with a significantly higher rate of MMR at 1 year compared with imatinib (pooled RR, 1.59; 95% CI, 1.44-1.75; *P* < .001). No statistical difference in overall survival at 1 year was found between new-generation TKIs and imatinib (pooled RR, 1.00; 95% CI, 1.00-1.01; *P* = .33).

**Conclusions and Relevance:**

When compared to imatinib, bosutinib, nilotinib, and ponatinib had higher relative risks of hepatotoxicity. Treatment with new-generation TKIs was associated with a higher MMR rate at 1 year but not with 1-year overall survival.

## Key Points

**Question:**

Are new-generation tyrosine kinase inhibitors associated with a higher risk of hepatotoxicity than imatinib in patients with chronic myeloid leukemia?

**Findings:**

In this meta-analysis of 9 studies including 3475 patients, bosutinib, nilotinib, and ponatinib were associated with an increased risk of hepatotoxicity compared with imatinib. No significant difference was found with dasatinib.

**Meaning:**

These findings suggest that hepatic function should be monitored frequently in patients treated with bosutinib, nilotinib, and ponatinib.

## Teaser

This systematic review and meta-analysis examines the relative risk of hepatotoxicity of new-generation BCR-ABL fusion oncoprotein tyrosine kinase inhibitors (TKIs) vs imatinib among patients with chronic myeloid leukemia.

## Introduction

Chronic myeloid leukemia (CML) is a myeloproliferative neoplasm with an incidence of 10 to 12 per 100 000 people worldwide.^1,2^ Currently, 5 tyrosine kinase inhibitors (TKIs) targeting the activated BCR-ABL1 oncoprotein have been approved by the US Food and Drug Administration (FDA) for the treatment of patients with CML.

Drug-induced hepatotoxicity is a major safety concern in clinical therapy, because nearly 50% of oral drugs on the market are associated with hepatotoxicity.^3^ Unfortunately, BCR-ABL TKIs are no exception.^4^ To date, more than 25 clinical cases of the first generation BCR-ABL TKI imatinib-induced hepatitis have been reported.^5,6^ As for the new-generation BCR-ABL TKIs, ponatinib therapy can cause rare instances of clinically apparent liver disease and even death,^7,8,9^ prompting the FDA to issue a boxed warning. Severe liver injury was also reported in patients with CML treated with nilotinib and dasatinib.^10,11^ However, among several studies comparing imatinib with the other BCR-ABL TKIs,^12,13,14,15,16,17,18,19^ only one mentions hepatic effects without stratification study.^19^ Hence, it is necessary to perform a meta-analysis focusing specifically on the relative risk of hepatotoxicity associated with each BCR-ABL TKI.

The aim of this study was to compare the risk of all grades and high grades (grades 3 and 4) hepatotoxicity associated with 4 new-generation BCR-ABL TKIs (bosutinib, dasatinib, nilotinib, and ponatinib) vs the first generation BCR-ABL TKI imatinib in patients with CML. Stratification by BCR-ABL TKI treatment was performed to provide drug-specific risk assessments. Overall survival (OS) and major molecular response (MMR) were also analyzed to provide global risk-benefit evaluations.

## Methods

### Definition of the Outcome

The primary outcome of interest was hepatotoxicity, including all grades and grades 3 and 4 of alanine aminotransferase (ALT) and aspartate aminotransferase (AST) elevation. Each variable of all grades and grades 3 and 4 was analyzed to compare the hepatotoxicity of a new generation of BCR-ABL TKI with that of imatinib. Adverse events were defined as the National Cancer Institute Common Terminology Criteria for Adverse Events, version 3.0 or 4.0. Data on the OS and the MMR reported at 12 months of TKI treatment were also extracted.

### Search Strategy and Study Selection

This study followed the Preferred Reporting Items for Systematic Reviews and Meta-analyses (PRISMA) reporting guideline.^20^ We searched the following databases using keywords and medical subject headings from January 2000 to April 2020: PubMed, Embase, and Cochrane library databases (eTable 1 in the Supplement). Only English language articles were considered. We also searched the publicly available trial register, ClinicalTrials.gov, to identify all clinical trials completed until April 2020. The inclusion and exclusion criteria have been previously published in the International Prospective Register of Systematic Reviews (PROSPERO: CRD42020202337).^21^ In this analysis, only randomized phase 2 or phase 3 clinical trials that compared bosutinib, dasatinib, nilotinib, or ponatinib with imatinib were included. If multiple publications of the same trial were identified, only the most recent and informative publication was selected. Study quality was assessed by Jadad score calculation for each study.^22^ This study did not require institutional review board approval nor was patient consent required, as the systematic review used published, publicly available data.

### Data Extraction

Data extraction was conducted independently by 2 investigators (Zhe Wang and X. Wang) and any disagreements were resolved by consensus. Data extracted for each study were: first author’s name, year of publication, trial phase, number of enrolled participants, drugs used in the experimental and standard arm, dosage, frequency, type of cancer, stage of disease, median age, sex, and data on ALT levels, AST levels, OS, and MMR. Some of these data were found at ClinicalTrials.gov to ensure data integrity. Race/ethnicity data were not collected because too many countries and regions were involved.

### Statistical Analysis

All calculations were performed using RevMan software version 5.3 (Cochrane Library). The *Q* statistic and *I^2^* statistic were applied to evaluate the heterogeneity among the various trials. When substantial heterogeneity was observed (*P* < .10 for *Q*-test or *I^2^* > 50%), the pooled estimate was calculated based on the random-effect model, otherwise, the fixed-effect model was used. The relative risk (RR) and 95% CI were calculated using the inverse variance method. Additionally, funnel plots and the Egger test were used to assess the publication bias of the enrolled studies. A 2-sided statistical test with *P* < .05 was considered significant. Statistical analysis was performed from April 2020 to March 2021.

## Results

### Study Selection

A total of 2666 records were retrieved initially for evaluation (PubMed: 812; Embase: 769; Cochrane library: 959; and ClinicalTrials.gov: 126), and 1743 remained after deduplication.^20^ After the first screening (reading titles and abstracts), 72 articles and abstracts fulfilled the previously established criteria and were evaluated in their entirety. Of these, 30 studies were identified as duplicate trials; 12 studies were conference abstracts and posters; another 12 were excluded because of the absence of hepatic toxicity data. Of remaining articles, 4 were single-group trials, and 2 were non-imatinib controlled trials. At the end of the selection procedure 3 studies were excluded because of the limited sample sizes. Finally, a total of 9 studies with 3475 patients were included for statistical analysis (eFigure 1 in the Supplement).^23,24,25,26,27,28,29,30,31^

### Characteristics of the Studies and Quality Assessment

The studies included in the meta-analysis were published between 2010 and 2018. TKIs included in the analysis were bosutinib (n = 2), dasatinib (n = 3), nilotinib (n = 3), and ponatinib (n = 1). As per inclusion criteria, all included studies were randomized, imatinib-controlled trials. Only one study was conducted in a single country (China), and 6 of 9 studies (67%) were conducted in the US or Canada in partnership with other countries. All 3475 patients included in this meta-analysis were diagnosed with the chronic phase of CML (CP CML); 2059 (59.2%) were male patients; and the median (range) patient age was 49 (18 to 91) years. Other characteristics and Jadad score of the included studies are shown in eTable 2 in the Supplement. Since most of the trials were not blinded, all included studies had low to moderate quality with a Jadad score of 2 or 3 (the Jadad score scale ranges from 0 to 5, with higher scores indicating higher study quality). Among all analyses performed, the random-effects model was used for all the primary outcomes, except for high-grades AST elevation, owing to presence of heterogeneity, and the fixed-effects model was used for the secondary outcomes. Funnel plots and the Egger test showed no publication bias (eFigure 2 in the Supplement). The sensitivity analysis indicated that removing studies one by one did not change the overall results (data not shown).

### Primary and Secondary Outcomes

The results of the fixed- or random-effects model RR for the elevation of ALT and AST levels, OS, and MMR were summarized in Table 1 and Table 2, respectively. The description of heterogeneity is also provided.

**Table 1.** RR of Hepatotoxicity Associated With BCR-ABL TKIs in the Treatment of CML

TKI	RR (95% CI)	*I^2^* statistic, %	*P* value
All grades of ALT elevation			
Overall	2.89 (1.78-4.69)	81	<.001
Bosutinib	4.27 (2.85-6.39)	31	<.001
Dasatinib	0.43 (0.11-1.67)	0	.22
Nilotinib	2.54 (1.26-5.11)	88	.009
Ponatinib	9.87 (2.35-41.50)	NA	.002
Grades 3-4 ALT elevation			
Overall	4.36 (2.00-9.50)	56	<.001
Bosutinib	7.91 (3.77-16.60)	33	<.001
Dasatinib	0.50 (0.09-2.71)	NA	.42
Nilotinib	2.94 (1.42-6.06)	0	.004
Ponatinib	14.81 (0.85-256.99)	NA	.06
All grades AST elevation			
Overall	2.20 (1.63-2.98)	51	<.001
Bosutinib	3.16 (2.27-4.39)	0	<.001
Dasatinib	0.60 (0.14-2.51)	0	.49
Nilotinib	1.82 (1.49-2.23)	0	<.001
Ponatinib	2.96 (1.21-7.26)	NA	.02
Grades 3-4 AST elevation			
Overall	2.65 (1.59-4.42)	25	<.001
Bosutinib	3.41 (1.85-6.27)	21	<.001
Dasatinib	0.67 (0.11-3.96)	NA	.66
Nilotinib	1.58 (0.49-5.09)	6	.44
Ponatinib	8.88 (0.48-163.60)	NA	.14

Abbreviations: AST, aspartate aminotransferase; ALT, alanine aminotransferase; CML, chronic myeloid leukemia; NA, not available; RR, relative risk; TKIs, tyrosine kinase inhibitors.

**Table 2.** RR of Major Molecular Response and Overall Survival Associated With BCR-ABL TKIs in the Treatment of CML

TKI	RR (95% CI)	*I^2^* statistic, %	*P* value
Overall survival			
Overall	1.00 (1.00-1.01)	44	.33
Bosutinib	1.01 (0.99-1.02)	51	.25
Dasatinib	1.00 (0.97-1.02)	80	.81
Nilotinib	1.00 (0.99-1.01)	0	.83
Ponatinib	1.01 (0.98-1.03)	NA	.55
Major molecular response			
Overall	1.59 (1.44-1.75)	14	<.001
Bosutinib	1.37 (1.16-1.61)	0	<.001
Dasatinib	1.60 (1.34-1.90)	0	<.001
Nilotinib	1.84 (1.56-2.19)	0	<.001
Ponatinib	2.08 (0.98-4.42)	NA	.06

Abbreviations: CML, chronic myeloid leukemia; NA, not available; RR, relative risk; TKIs, tyrosine kinase inhibitors.

### ALT Elevation

**Figure 1.**
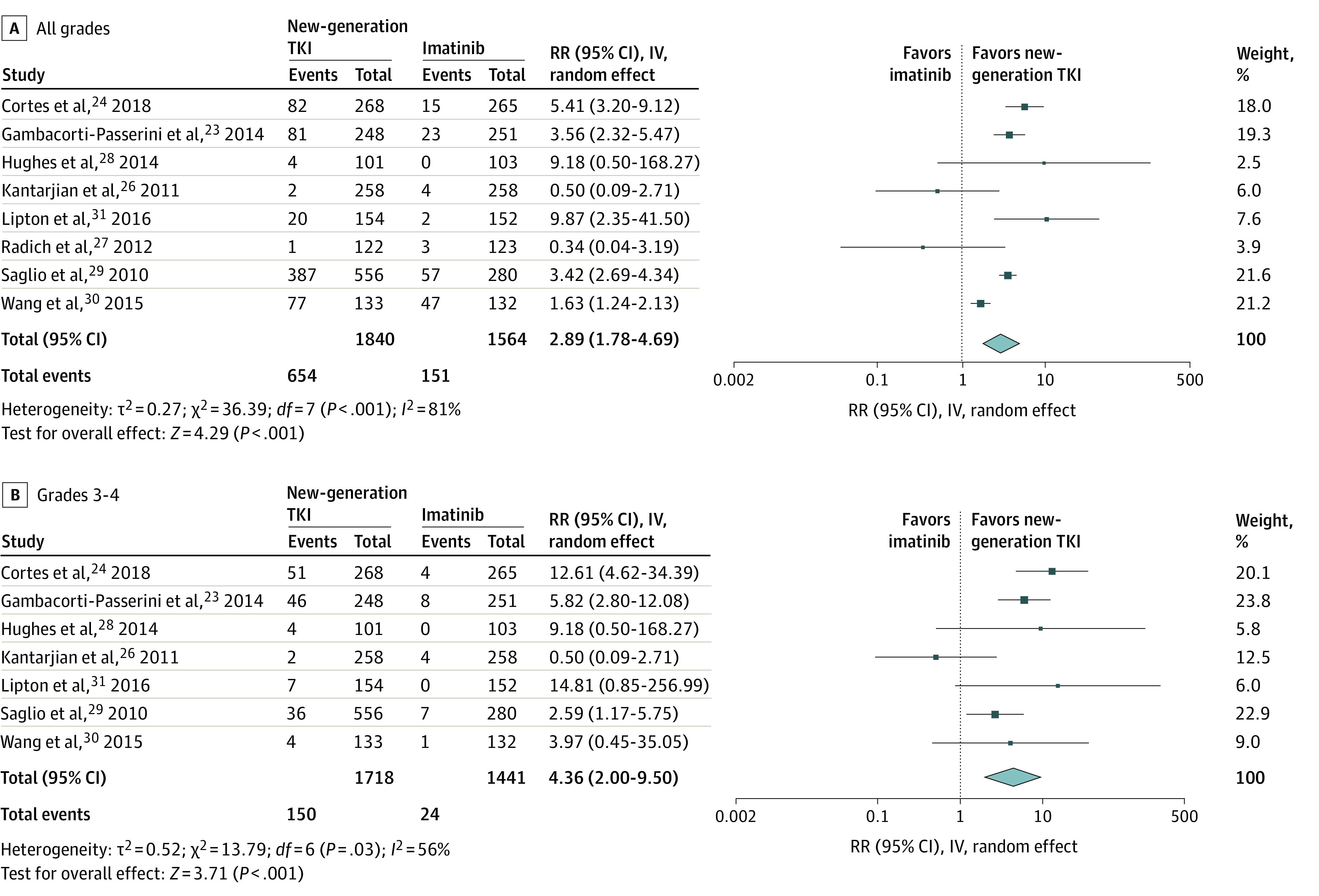
Pooled Analysis of All-Grade and Grades 3-4 Alanine Aminotransferase Elevation IV indicates inverse variance method; RR, relative risk; TKI, tyrosine kinase inhibitors.

All grades of ALT elevation occurred in 654 of 1840 patients (35.5%) treated with new-generation TKIs vs 151 of 1564 patients (9.7%) treated with imatinib. The use of new-generation TKIs was associated with a statistically significant overall increase in the risk of developing all grades of ALT elevation compared with imatinib (RR, 2.89; 95% CI, 1.78-4.69; *P* < .001) (Figure 1A). When stratified by type of drug, bosutinib (RR, 4.27; 95% CI, 2.85-6.39; *P* < .001), nilotinib (RR, 2.54; 95% CI, 1.26-5.11; *P* = .009), and ponatinib (RR, 9.87; 95% CI, 2.35-41.50; *P* = .002) were associated with an increased risk of all grades of ALT elevation, but dasatinib was not associated with an increased risk of all grades of ALT elevation (RR, 0.43; 95% CI, 0.11-1.67; *P* = .22).

Grades 3 and 4 ALT elevation was observed in 150 of 1718 patients (8.73%) treated with new-generation TKIs compared with 24 of 1440 patients (1.67%) treated with imatinib. Patients who received new-generation TKIs were more likely to develop grades 3 and 4 elevation of ALT levels (RR, 4.36; 95% CI, 2.00-9.50; *P* < .001) compared with controls (Figure 1B). Subgroup analysis indicated that bosutinib (RR, 7.91; 95% CI, 3.77-16.60; *P* < .001) and nilotinib (RR, 2.94; 95% CI, 1.42-6.06; *P* < .001) were associated with an increased risk of grades 3 and 4 ALT elevation, whereas dasatinib (RR, 0.50; 95% CI, 0.09-2.71; *P* = .42) and ponatinib (RR, 14.81; 95% CI, 0.85-256.99; *P* = .06) were not.

### AST Elevation

**Figure 2.**
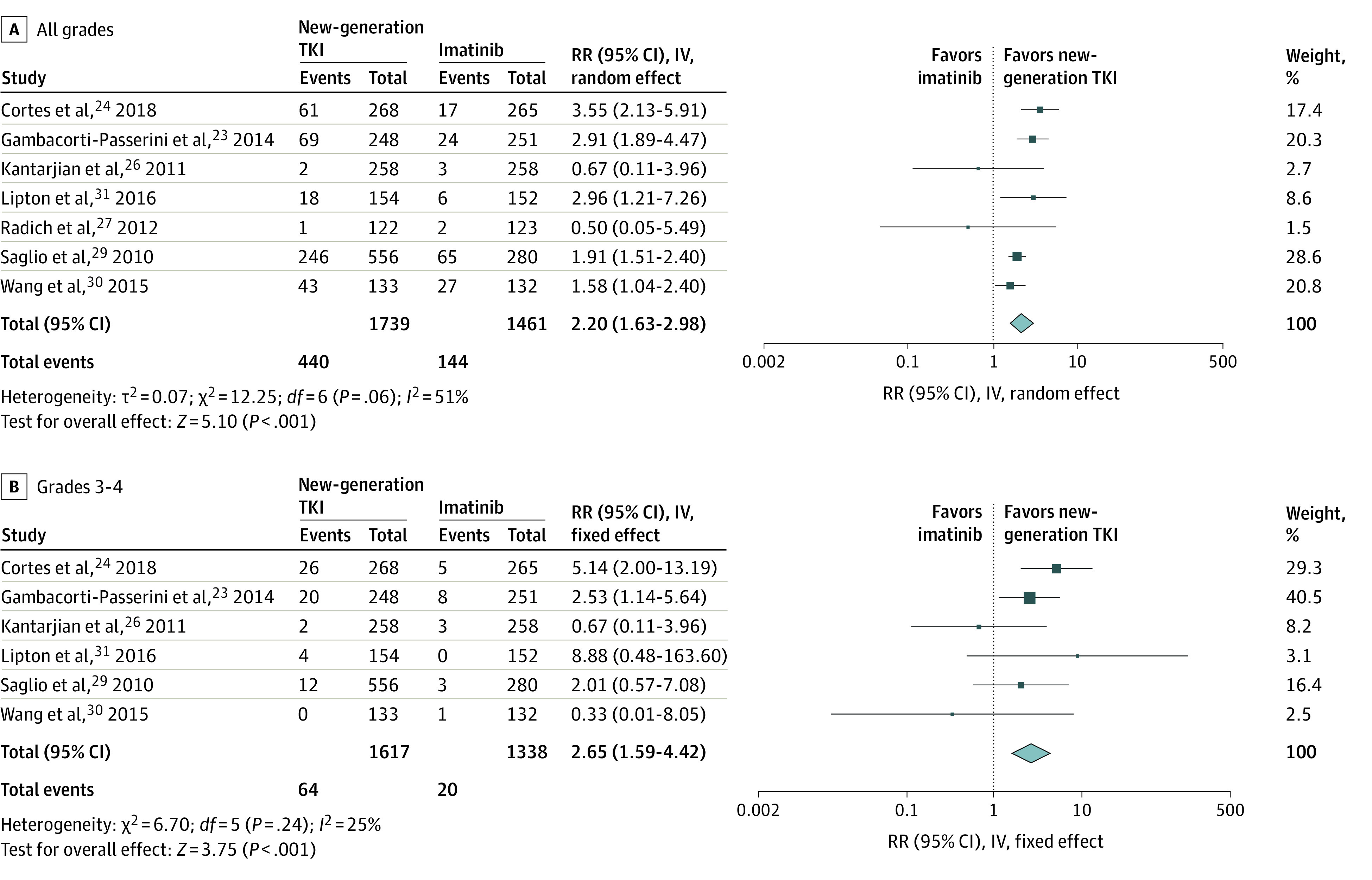
Pooled Analysis of All-Grade and Grades 3-4 Aspartate Aminotransferase Elevation IV indicates inverse variance method; RR, relative risk; TKI, tyrosine kinase inhibitors.

All grades of AST elevation occurred in 440 of 1739 patients (25.3%) treated with new-generation TKIs vs 144 of 1461 patients (9.9%) treated with imatinib. New-generation TKIs were also associated with a significantly increased risk of all grades of AST elevation compared with imatinib (RR, 2.20; 95% CI, 1.63-2.98; *P* < .001) (Figure 2A). Subgroup analysis indicated that the difference was statistically significant for bosutinib (RR, 3.16; 95% CI, 2.27-4.39; *P* < .001), nilotinib (RR, 1.82; 95% CI, 1.49-2.23; *P* < .001), and ponatinib (RR, 2.96; 95% CI, 1.21-7.26; *P* = .02), whereas no difference was found for dasatinib (RR, 0.60; 95% CI, 0.14-2.51; *P* = .49).

Grades 3 and 4 AST elevation was observed in 64 of 1617 patients (3.9%) treated with new-generation TKIs vs 20 of 1338 patients (1.5%) treated with imatinib. Compared with imatinib, new-generation TKIs were associated with a significantly increased risk of high-grade AST elevation (RR, 2.65; 95% CI, 1.59-4.42; *P* < .001) (Figure 2B). Significantly increased risks were observed for bosutinib (RR, 3.41; 95% CI, 1.85-6.27; *P* < .001) but not the other TKIs (dasatinib: RR, 0.67; 95% CI, 0.11-3.96; *P* = .66; nilotinib: RR, 1.58; 95% CI, 0.49-5.09; *P* = .44; and ponatinib: RR, 8.88; 95% CI, 0.48-163.6; *P* = .14).

### Overall Survival

**Figure 3.**
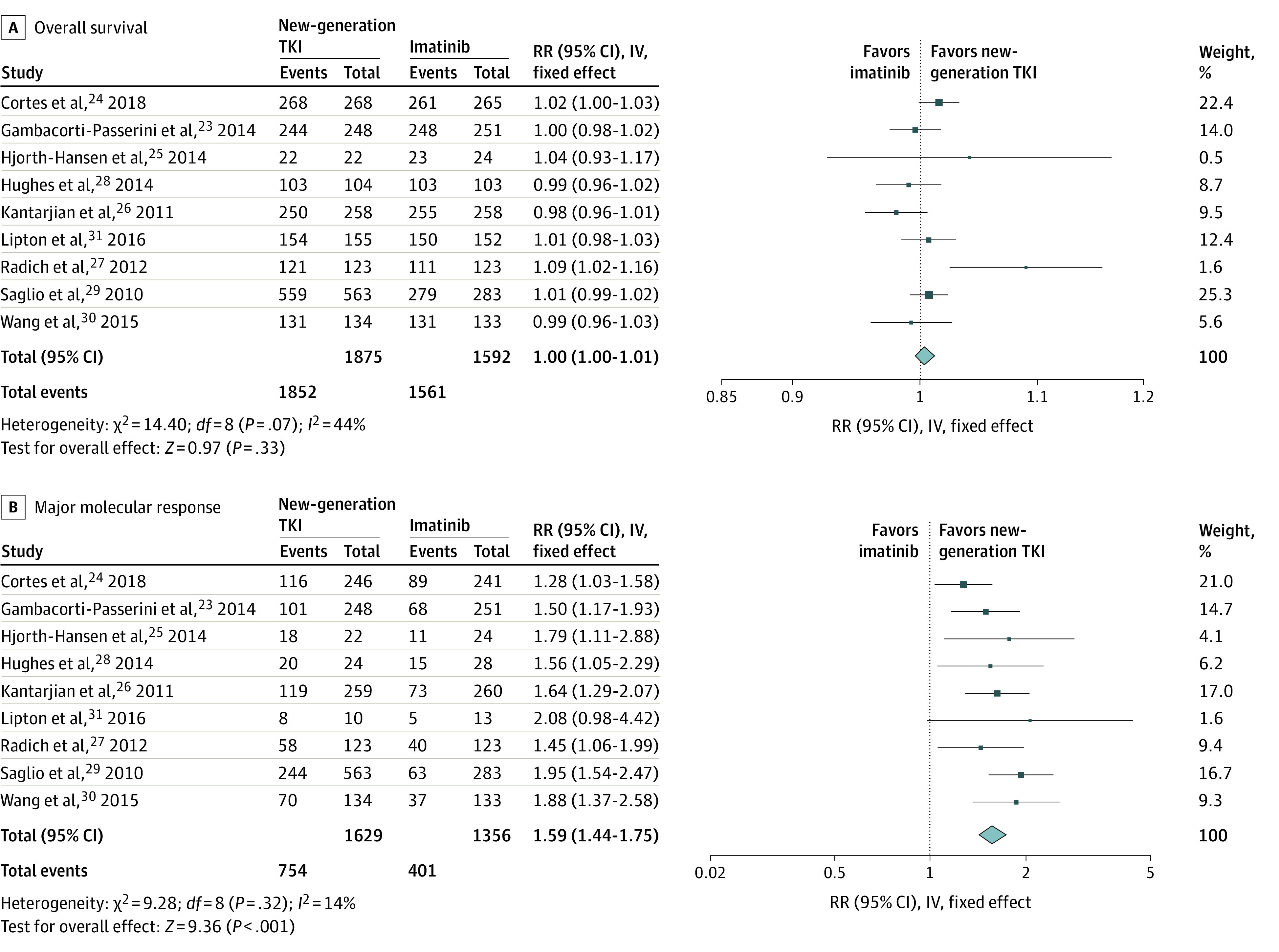
Pooled Analysis of Overall Survival and Major Molecular Response IV indicates inverse variance method; RR, relative risk; TKI, tyrosine kinase inhibitors.

As shown in Figure 3A, death during the first year occurred in 23 of 1875 patients (1.23%) treated with new-generation TKIs compared with 31 of 1592 patients (1.95%) treated with imatinib. There was no statistical difference in mortality rate at 1 year between new-generation TKIs and imatinib (RR, 1.00; 95% CI, 1.00-1.01; *P* = .33). Stratification by treatment did not change the results (bosutinib: RR, 1.01; 95% CI, 0.99-1.02; *P* = .25; dasatinib, RR, 1.00; 95% CI, 0.97-1.02; *P* = .81; nilotinib: RR, 1.00; 95% CI, 0.99-1.01; *P* = .83; and ponatinib: RR, 1.01; 95% CI, 0.98-1.03; *P* = .55).

### MMR

As shown in Figure 3B, 754 of 1629 patients (46.3%) treated with new-generation TKIs achieved an MMR at 1 year compared with 401 of 1356 patients (29.6%) treated with imatinib. Pooled data showed that new-generation TKIs were associated with a higher rate of MMR at 1 year compared with imatinib (RR, 1.59; 95% CI, 1.44-1.75; *P* < .001). Similar results were observed for each TKI, although the increase of MMR rate for ponatinib did not achieve statistical significance (bosutinib: RR, 1.37; 95% CI, 1.16-1.61; *P* < .001; dasatinib: RR, 1.60; 95% CI, 1.34-1.90; *P* < .001; nilotinib: RR, 1.84; 95% CI, 1.56-2.19; *P* < .001; and ponatinib: RR, 2.08; 95% CI, 0.98-4.42; *P* = .06).

## Discussion

Hepatotoxicity is one of the serious safety issues signaled in preapproval clinical trials with TKIs. The FDA has approved more than 60 TKIs for human use as of June 2020, with 5 having black box warnings for hepatotoxicity in product labeling (lapatinib, pazopanib, sunitinib, regorafenib, and ponatinib). Clinical trials indicated a low-grade elevation in serum liver enzyme (ALT and/or AST) in 25% to 30% and a high-grade elevation in approximately 2% of patients treated with TKIs.^32^ Previously published meta-analyses also found that the use of vascular endothelial growth factor receptor (VEGFR) TKIs, epidermal growth factor receptor (EGFR) TKIs, and anaplastic lymphoma kinase (ALK) TKIs significantly increased the risk of developing liver toxicities.^33,34,35,36^ A recent meta-analysis indicated that patients treated with new-generation BCR-ABL TKIs were associated with more frequent hepatic events than imatinib,^19^ but it did not make stratified meta-analyses of these drugs.

To our knowledge, this is the first meta-analysis focusing specifically on hepatotoxicity associated with new-generation TKIs vs the first generation TKI imatinib in patients with CML. Overall survival and efficacy were also analyzed to provide global risk-benefit evaluation. We were able to demonstrate that the treatment with bosutinib, nilotinib, or ponatinib was associated with a higher risk of hepatotoxicity than that of imatinib, whereas no significant increased risks were found for dasatinib. New-generation TKIs were associated with improvement in the rate of MMR but not for 1-year overall survival.

The precise mechanism of TKI-related hepatotoxicity is still not well clarified. It has been hypothesized that the formation of reactive metabolites during metabolism plays a key role in TKI-induced hepatotoxicity.^37^ It has been demonstrated previously that cytochrome P450-mediated bioactivation of gefitinib, erlotinib, lapatinib, pazopanib, and sunitinib can generate reactive quinone-imine and epoxide compounds.^38,39,40,41,42^ These reactive metabolites can covalently bind to nucleophilic macromolecules in target cells such as endogenous protein, lipids, or DNA, causing irreversible cellular damage and even cell death. Immune mediated response and drug metabolizing enzyme-based drug-drug interactions have also been proposed to be a mechanism of toxicity.^43,44^ Although little is known about the underlying mechanisms of BCR-ABL TKIs–induced liver injury, it has been reported that the direct and indirect mitochondrial toxicity may represent a toxicological mechanism of dasatinib and ponatinib.^45,46^ Furthermore, UGT1A1 is the sole physiologically relevant enzyme participating in the metabolic elimination of bilirubin, an endogenous toxic metabolite. The inhibition of UGT1A1 may lead to accumulation of bilirubin to toxic levels, which may be the mechanism of atazanavir-, indinavir-, erlotinib-, and nilotinib-related hepatic toxicities (eg, jaundice and hyperbilirubinemia).^47,48,49^ Further studies will be needed to investigate the mechanism of BCR-ABL TKIs–associated hepatotoxicity.

To provide a personalized medication strategy, the physician should consider the patient’s characteristics and the efficacy and safety profiles of the different TKIs. Imatinib, nilotinib, and dasatinib are currently registered for the first-line treatment of CP CML.^50^ For older patients with the goal of the treatment of improving survival, imatinib is the first choice. If intolerance or resistance occurs, it is recommended to switch to dasatinib. For patients with newly diagnosed CP CML without baseline hepatic impairment comorbidities and for whom achieving a deep molecular response is the predominant objective, dasatinib remains to be an excellent choice. In cases of treatment failure, bosutinib and nilotinib are preferred. Particularly, the use of bosutinib and nilotinib as first-line treatments requires a screening for potential risk factors (eg, serum transaminases). Ponatinib should be reserved for patients with advanced disease, with the *T315I* variant, or for whom other treatments cannot be used.

### Limitations

A limitation in this analysis concerns the lack of access to individual data that might have provided important additional information, including the number of deaths due to liver failure and the baseline hepatic risk factors in each study group. Reporting of hepatic adverse events was also lacking in many studies, and the exclusion of these studies might have introduced bias into the analysis. Moreover, apart from elevation of ALT and AST, other biochemical markers of liver injury include bilirubin, alkaline phosphatase, albumin concentration, and the prothrombin time should be further analyzed to improve sensitivity or specificity of the evaluation of liver injury. Furthermore, the exclusive reliance on English-language studies may not represent all the evidence.

## Conclusions

This meta-analysis found a significant increase in the risk of hepatotoxicity associated with the use of bosutinib, nilotinib, and ponatinib compared with imatinib. Treatment with these TKIs should be considered with frequent hepatic function monitoring. As the risk of hepatotoxicity of nilotinib seems to be associated with the dose administered,^29^ further studies are needed to clearly define the dose regimen of each BCR-ABL TKI, which will provide the best clinically relevant benefit-risk profile.

## Notes

Supplement. **eTable 1.** Search Strategy **eTable 2.** Characteristics of Included Studies and Quality Assessment **eFigure 1.** Flow Diagram of Study Selection According to PRISMA Statement **eFigure 2.** Evaluation of Publications Bias
