# Supplementary material for: Comparison of Hepatotoxicity Associated With New BCR-ABL Tyrosine Kinase Inhibitors vs Imatinib Among Patients With Chronic Myeloid Leukemia: A Systematic Review and Meta-analysis
Source: JAMA Netw Open. 2021 Jul 22;4(7):e2120165. doi: 10.1001/jamanetworkopen.2021.20165 (PMC8299317; doi:10.1001/jamanetworkopen.2021.20165)
Supplement: Supplement. — eTable 1. Search Strategy eTable 2. Characteristics of Included Studies and Quality Assessment eFigure 1. Flow Diagram of Study Selection According to PRISMA Statement eFigure 2. Evaluation of Publications Bias [file jamanetwopen-e2120165-s001.pdf]

Supplemental Online Content

Wang Z, Wang X, Wang Z, et al. Comparison of hepatotoxicity associated with new BCR-ABL tyrosine kinase inhibitors vs imatinib among patients with chronic myeloid leukemia: a systematic review and meta-analysis. *JAMA Netw Open.* 2021;4(7):e2120165. doi:10.1001/jamanetworkopen.2021.20165

eTable 1. Search Strategy

eTable 2. Characteristics of Included Studies and Quality Assessment

eFigure 1. Flow Diagram of Study Selection According to PRISMA Statement

eFigure 2. Evaluation of Publications Bias

This supplemental material has been provided by the authors to give readers additional information about their work.

© 2021 Wang Z et al. *JAMA Network Open.*

eTable 1: Search strategy (January 2000 to April 2020)

Database	Drug	Search Strategy	Number of hits
PubMed/ Embase	Bosutinib	Search ((((bosutinib[MeSH Major Topic]) OR SKI606) OR SKI-606)) AND ((Leukemia, Myelogenous, Chronic, BCR-ABL Positive[MeSH Major Topic]) OR Leukemia, Chronic Myelogenous) OR Leukemia, Chronic Myeloid) OR Leukemia, Granulocytic, Chronic) OR Leukemia, Myelocytic, Chronic) OR Leukemia, Myelogenous, Chronic) OR Leukemia, Myelogenous, Ph1 Positive) OR Leukemia, Myelogenous, Ph1-Positive) OR Leukemia, Myeloid, Chronic) OR Leukemia, Myeloid, Ph1 Positive) OR Leukemia, Myeloid, Ph1-Positive) OR Leukemia, Myeloid, Philadelphia Positive) OR Leukemia, Myeloid, Philadelphia-Positive) OR Myelocytic Leukemia, Chronic) OR Chronic Myelocytic Leukemia) OR Chronic Myelocytic Leukemias) OR Leukemia, Chronic Myelocytic) OR Leukemias, Chronic Myelocytic) OR Myelocytic Leukemias, Chronic) OR Myelogenous Leukemia, Chronic) OR Chronic Myelogenous Leukemia) OR Chronic Myelogenous Leukemias) OR Leukemias, Chronic Myelogenous) OR Myelogenous Leukemias, Chronic) OR Myelogenous Leukemia, Ph1-Positive) OR Leukemia, Ph1-Positive Myelogenous) OR Leukemias, Ph1-Positive Myelogenous) OR Myelogenous Leukemia, Ph1 Positive) OR Myelogenous Leukemias, Ph1-Positive) OR Ph1-Positive Myelogenous Leukemia) OR Ph1-Positive Myelogenous Leukemias) OR Myeloid Leukemia, Chronic) OR Chronic Myeloid Leukemia) OR Chronic Myeloid Leukemias) OR Leukemias, Chronic Myeloid) OR Myeloid Leukemias, Chronic) OR Myeloid Leukemia, Ph1-Positive) OR Leukemia, Ph1-Positive Myeloid) OR Leukemias, Ph1-Positive Myeloid) OR Myeloid Leukemia, Ph1 Positive) OR Myeloid Leukemias, Ph1-Positive) OR Ph1-Positive Myeloid Leukemia) OR Ph1-Positive Myeloid Leukemias) OR Myeloid Leukemia, Philadelphia-Positive) OR Leukemia, Philadelphia-Positive Myeloid) OR Leukemias, Philadelphia-Positive Myeloid) OR Myeloid Leukemia, Philadelphia Positive) OR Myeloid Leukemias, Philadelphia-Positive) OR Philadelphia-Positive Myeloid	67

© 2021 Wang Z et al. *JAMA Network Open.*

		Leukemia) OR Philadelphia-Positive Myeloid Leukemias) OR Granulocytic Leukemia, Chronic) OR Chronic Granulocytic Leukemia) OR Chronic Granulocytic Leukemias) OR Granulocytic Leukemias, Chronic) OR Leukemia, Chronic Granulocytic) OR Leukemias, Chronic Granulocytic) Filters: Clinical Trial	
	Dasatinib	Search (((((((dasatinib[MeSH Major Topic]) OR Sprycel) OR BMS 354825) OR 354825, BMS) OR BMS354825) OR BMS-354825)) AND ((Leukemia, Myelogenous, Chronic, BCR-ABL Positive[MeSH Major Topic]) OR Leukemia, Chronic Myelogenous) OR Leukemia, Chronic Myeloid) OR Leukemia, Granulocytic, Chronic) OR Leukemia, Myelocytic, Chronic) OR Leukemia, Myelogenous, Chronic) OR Leukemia, Myelogenous, Ph1 Positive) OR Leukemia, Myelogenous, Ph1-Positive) OR Leukemia, Myeloid, Chronic) OR Leukemia, Myeloid, Ph1 Positive) OR Leukemia, Myeloid, Ph1-Positive) OR Leukemia, Myeloid, Philadelphia Positive) OR Leukemia, Myeloid, Philadelphia-Positive) OR Myelocytic Leukemia, Chronic) OR Chronic Myelocytic Leukemia) OR Chronic Myelocytic Leukemias) OR Leukemia, Chronic Myelocytic) OR Leukemias, Chronic Myelocytic) OR Myelocytic Leukemias, Chronic) OR Myelogenous Leukemia, Chronic) OR Chronic Myelogenous Leukemia) OR Chronic Myelogenous Leukemias) OR Leukemias, Chronic Myelogenous) OR Myelogenous Leukemias, Chronic) OR Myelogenous Leukemia, Ph1-Positive) OR Leukemia, Ph1-Positive Myelogenous) OR Leukemias, Ph1-Positive Myelogenous) OR Myelogenous Leukemia, Ph1 Positive) OR Myelogenous Leukemias, Ph1-Positive) OR Ph1-Positive Myelogenous Leukemia) OR Ph1-Positive Myelogenous Leukemias) OR Myeloid Leukemia, Chronic) OR Chronic Myeloid Leukemia) OR Chronic Myeloid Leukemias) OR Leukemias, Chronic Myeloid) OR Myeloid Leukemias, Chronic) OR Myeloid Leukemia, Ph1-Positive) OR Leukemia, Ph1-Positive Myeloid) OR Leukemias, Ph1-Positive Myeloid) OR Myeloid Leukemia, Ph1 Positive) OR Myeloid Leukemias, Ph1-Positive) OR Ph1-Positive Myeloid Leukemia) OR Ph1-Positive Myeloid Leukemias) OR Myeloid Leukemia, Philadelphia-Positive) OR Leukemia, Philadelphia-Positive Myeloid) OR Leukemias, Philadelphia-Positive Myeloid) OR Myeloid Leukemia, Philadelphia Positive) OR Myeloid Leukemias, Philadelphia-Positive) OR Philadelphia-Positive Myeloid Leukemia) OR Philadelphia-Positive Myeloid Leukemias) OR	140

© 2021 Wang Z et al. *JAMA Network Open.*

		Granulocytic Leukemia, Chronic) OR Chronic Granulocytic Leukemia) OR Chronic Granulocytic Leukemias) OR Granulocytic Leukemias, Chronic) OR Leukemia, Chronic Granulocytic) OR Leukemias, Chronic Granulocytic) Filters: Clinical Trial	
	Nilotinib	Search ((((nilotinib[MeSH Major Topic]) OR Tasigna) OR AMN107)) AND (((Leukemia, Myelogenous, Chronic, BCR-ABL Positive[MeSH Major Topic]) OR Leukemia, Chronic Myelogenous) OR Leukemia, Chronic Myeloid) OR Leukemia, Granulocytic, Chronic) OR Leukemia, Myelocytic, Chronic) OR Leukemia, Myelogenous, Chronic) OR Leukemia, Myelogenous, Ph1 Positive) OR Leukemia, Myelogenous, Ph1-Positive) OR Leukemia, Myeloid, Chronic) OR Leukemia, Myeloid, Ph1 Positive) OR Leukemia, Myeloid, Ph1-Positive) OR Leukemia, Myeloid, Philadelphia Positive) OR Leukemia, Myeloid, Philadelphia-Positive) OR Myelocytic Leukemia, Chronic) OR Chronic Myelocytic Leukemia) OR Chronic Myelocytic Leukemias) OR Leukemia, Chronic Myelocytic) OR Leukemias, Chronic Myelocytic) OR Myelocytic Leukemias, Chronic) OR Myelogenous Leukemia, Chronic) OR Chronic Myelogenous Leukemia) OR Chronic Myelogenous Leukemias) OR Leukemias, Chronic Myelogenous) OR Myelogenous Leukemias, Chronic) OR Myelogenous Leukemia, Ph1-Positive) OR Leukemia, Ph1-Positive Myelogenous) OR Leukemias, Ph1-Positive Myelogenous) OR Myelogenous Leukemia, Ph1 Positive) OR Myelogenous Leukemias, Ph1-Positive) OR Ph1-Positive Myelogenous Leukemia) OR Ph1-Positive Myelogenous Leukemias) OR Myeloid Leukemia, Chronic) OR Chronic Myeloid Leukemia) OR Chronic Myeloid Leukemias) OR Leukemias, Chronic Myeloid) OR Myeloid Leukemias, Chronic) OR Myeloid Leukemia, Ph1-Positive) OR Leukemia, Ph1-Positive Myeloid) OR Leukemias, Ph1-Positive Myeloid) OR Myeloid Leukemia, Ph1 Positive) OR Myeloid Leukemias, Ph1-Positive) OR Ph1-Positive Myeloid Leukemia) OR Ph1-Positive Myeloid Leukemias) OR Myeloid Leukemia, Philadelphia-Positive) OR Leukemia, Philadelphia-Positive Myeloid) OR Leukemias, Philadelphia-Positive Myeloid) OR Myeloid Leukemia, Philadelphia Positive) OR Myeloid Leukemias, Philadelphia-Positive) OR Philadelphia-Positive Myeloid Leukemia) OR Philadelphia-Positive Myeloid Leukemias) OR Granulocytic Leukemia, Chronic) OR Chronic Granulocytic	262

© 2021 Wang Z et al. *JAMA Network Open*.

		Leukemia) OR Chronic Granulocytic Leukemias) OR Granulocytic Leukemias, Chronic) OR Leukemia, Chronic Granulocytic) OR Leukemias, Chronic Granulocytic) Filters: Clinical Trial	
	Ponatinib	Search ((((((ponatinib[MeSH Major Topic]) OR ponatinib hydrochloride) OR AP24534) OR AP-24534) OR AP 24534)) AND ((Leukemia, Myelogenous, Chronic, BCR-ABL Positive[MeSH Major Topic]) OR Leukemia, Chronic Myelogenous) OR Leukemia, Chronic Myeloid) OR Leukemia, Granulocytic, Chronic) OR Leukemia, Myelocytic, Chronic) OR Leukemia, Myelogenous, Chronic) OR Leukemia, Myelogenous, Ph1 Positive) OR Leukemia, Myelogenous, Ph1-Positive) OR Leukemia, Myeloid, Chronic) OR Leukemia, Myeloid, Ph1 Positive) OR Leukemia, Myeloid, Ph1-Positive) OR Leukemia, Myeloid, Philadelphia Positive) OR Leukemia, Myeloid, Philadelphia-Positive) OR Myelocytic Leukemia, Chronic) OR Chronic Myelocytic Leukemia) OR Chronic Myelocytic Leukemias) OR Leukemia, Chronic Myelocytic) OR Leukemias, Chronic Myelocytic) OR Myelocytic Leukemias, Chronic) OR Myelogenous Leukemia, Chronic) OR Chronic Myelogenous Leukemia) OR Chronic Myelogenous Leukemias) OR Leukemias, Chronic Myelogenous) OR Myelogenous Leukemias, Chronic) OR Myelogenous Leukemia, Ph1-Positive) OR Leukemia, Ph1-Positive Myelogenous) OR Leukemias, Ph1-Positive Myelogenous) OR Myelogenous Leukemia, Ph1 Positive) OR Myelogenous Leukemias, Ph1-Positive) OR Ph1-Positive Myelogenous Leukemia) OR Ph1-Positive Myelogenous Leukemias) OR Myeloid Leukemia, Chronic) OR Chronic Myeloid Leukemia) OR Chronic Myeloid Leukemias) OR Leukemias, Chronic Myeloid) OR Myeloid Leukemias, Chronic) OR Myeloid Leukemia, Ph1-Positive) OR Leukemia, Ph1-Positive Myeloid) OR Leukemias, Ph1-Positive Myeloid) OR Myeloid Leukemia, Ph1 Positive) OR Myeloid Leukemias, Ph1-Positive) OR Ph1-Positive Myeloid Leukemia) OR Ph1-Positive Myeloid Leukemias) OR Myeloid Leukemia, Philadelphia-Positive) OR Leukemia, Philadelphia-Positive Myeloid) OR Leukemias, Philadelphia-Positive Myeloid) OR Myeloid Leukemia, Philadelphia Positive) OR Myeloid Leukemias, Philadelphia-Positive) OR Philadelphia-Positive Myeloid Leukemia) OR Philadelphia-Positive Myeloid Leukemias) OR Granulocytic Leukemia, Chronic) OR Chronic Granulocytic Leukemia) OR Chronic Granulocytic Leukemias) OR Granulocytic	35

© 2021 Wang Z et al. *JAMA Network Open.*

		Leukemias, Chronic) OR Leukemia, Chronic Granulocytic) OR Leukemias, Chronic Granulocytic) Filters: Clinical Trial	
	Imatinib	Search ((((((((((((((((Imatinib Mesylate[MeSH Major Topic]) OR Mesylate, Imatinib) OR Imatinib Methanesulfonate) OR Methanesulfonate, Imatinib) OR STI571) OR STI-571) OR STI 571) OR Gleevec) OR Glivec) OR ST 1571) OR ST1571) OR CGP 57148) OR CGP57148B) OR CGP-57148) OR CGP57148) OR Imatinib)) AND ((Leukemia, Myelogenous, Chronic, BCR-ABL Positive[MeSH Major Topic]) OR Leukemia, Chronic Myelogenous) OR Leukemia, Chronic Myeloid) OR Leukemia, Granulocytic, Chronic) OR Leukemia, Myelocytic, Chronic) OR Leukemia, Myelogenous, Chronic) OR Leukemia, Myelogenous, Ph1 Positive) OR Leukemia, Myelogenous, Ph1-Positive) OR Leukemia, Myeloid, Chronic) OR Leukemia, Myeloid, Ph1 Positive) OR Leukemia, Myeloid, Ph1-Positive) OR Leukemia, Myeloid, Philadelphia Positive) OR Leukemia, Myeloid, Philadelphia-Positive) OR Myelocytic Leukemia, Chronic) OR Chronic Myelocytic Leukemia) OR Chronic Myelocytic Leukemias) OR Leukemia, Chronic Myelocytic) OR Leukemias, Chronic Myelocytic) OR Myelocytic Leukemias, Chronic) OR Myelogenous Leukemia, Chronic) OR Chronic Myelogenous Leukemia) OR Chronic Myelogenous Leukemias) OR Leukemias, Chronic Myelogenous) OR Myelogenous Leukemias, Chronic) OR Myelogenous Leukemia, Ph1-Positive) OR Leukemia, Ph1-Positive Myelogenous) OR Leukemias, Ph1-Positive Myelogenous) OR Myelogenous Leukemia, Ph1 Positive) OR Myelogenous Leukemias, Ph1-Positive) OR Ph1-Positive Myelogenous Leukemia) OR Ph1-Positive Myelogenous Leukemias) OR Myeloid Leukemia, Chronic) OR Chronic Myeloid Leukemia) OR Chronic Myeloid Leukemias) OR Leukemias, Chronic Myeloid) OR Myeloid Leukemias, Chronic) OR Myeloid Leukemia, Ph1-Positive) OR Leukemia, Ph1-Positive Myeloid) OR Leukemias, Ph1-Positive Myeloid) OR Myeloid Leukemia, Ph1 Positive) OR Myeloid Leukemias, Ph1-Positive) OR Ph1-Positive Myeloid Leukemia) OR Ph1-Positive Myeloid Leukemias) OR Myeloid Leukemia, Philadelphia-Positive) OR Leukemia, Philadelphia-Positive Myeloid) OR Leukemias, Philadelphia-Positive Myeloid) OR Myeloid Leukemia, Philadelphia Positive) OR Myeloid Leukemias, Philadelphia-Positive) OR Philadelphia-Positive Myeloid	1077

© 2021 Wang Z et al. *JAMA Network Open.*

		Leukemia) OR Philadelphia-Positive Myeloid Leukemias) OR Granulocytic Leukemia, Chronic) OR Chronic Granulocytic Leukemia) OR Chronic Granulocytic Leukemias) OR Granulocytic Leukemias, Chronic) OR Leukemia, Chronic Granulocytic) OR Leukemias, Chronic Granulocytic) Filters: Clinical Trial	
Cochrane Library	Bosutinib	#1 (Bosutinib):ti,ab,kw OR (SKI606):ti,ab,kw OR (SKI-606):ti,ab,kw (Word variations have been searched)<br>#2 (Leukemia, Myelogenous, Chronic, BCR-ABL Positive):ti,ab,kw OR (Philadelphia-Positive Myeloid Leukemia):ti,ab,kw OR (Myeloid Leukemias, Chronic):ti,ab,kw OR (Leukemia, Myelogenous, Ph1 Positive):ti,ab,kw OR (Leukemias, Chronic Granulocytic):ti,ab,kw (Word variations have been searched)<br>#3 (Leukemia, Myelogenous, Chronic, BCR-ABL Positive):ti,ab,kw OR (Myelogenous Leukemias, Ph1 Positive):ti,ab,kw OR (Leukemia, Myeloid, Philadelphia Positive):ti,ab,kw OR (Myelocytic Leukemias, Chronic):ti,ab,kw OR (Granulocytic Leukemia, Chronic):ti,ab,kw (Word variations have been searched)<br>#4 (Chronic Myelogenous Leukemia):ti,ab,kw OR (Leukemias, Ph1 Positive Myelogenous):ti,ab,kw OR (Myelogenous Leukemias, Chronic):ti,ab,kw OR (Chronic Granulocytic Leukemia):ti,ab,kw OR (Leukemia, Myelogenous, Ph1 Positive):ti,ab,kw (Word variations have been searched)<br>#5 (Leukemia, Philadelphia-Positive Myeloid):ti,ab,kw OR (Ph1 Positive Myeloid Leukemia):ti,ab,kw OR (Myeloid Leukemia, Chronic):ti,ab,kw OR (Leukemia, Myeloid, Ph1 Positive):ti,ab,kw OR (Leukemia, Granulocytic, Chronic):ti,ab,kw (Word variations have been searched)<br>#6 (Leukemia, Ph1 Positive Myeloid):ti,ab,kw OR (Chronic Myelocytic Leukemia):ti,ab,kw OR (Myeloid Leukemias, Ph1 Positive):ti,ab,kw OR (Granulocytic Leukemias, Chronic):ti,ab,kw OR (Myeloid Leukemia, Philadelphia Positive):ti,ab,kw (Word variations have been searched)<br>#7 #2 or #3 or #4 or #5 or #6<br>#8 #1 and #7	82
	Dasatinib	#1 (dasatinib):ti,ab,kw OR (Sprycel):ti,ab,kw OR (BMS-354825):ti,ab,kw OR (BMS354825):ti,ab,kw OR (354825, BMS):ti,ab,kw (Word variations have been searched)<br>#2 (Leukemia, Myelogenous, Chronic, BCR-ABL Positive):ti,ab,kw OR (Philadelphia-Positive Myeloid	244

© 2021 Wang Z et al. *JAMA Network Open.*

		Leukemia):ti,ab,kw OR (Myeloid Leukemias, Chronic):ti,ab,kw OR (Leukemia, Myelogenous, Ph1 Positive):ti,ab,kw OR (Leukemias, Chronic Granulocytic):ti,ab,kw (Word variations have been searched)<br>#3 (Leukemia, Myelogenous, Chronic, BCR-ABL Positive):ti,ab,kw OR (Myelogenous Leukemias, Ph1 Positive):ti,ab,kw OR (Leukemia, Myeloid, Philadelphia Positive):ti,ab,kw OR (Myelocytic Leukemias, Chronic):ti,ab,kw OR (Granulocytic Leukemia, Chronic):ti,ab,kw (Word variations have been searched)<br>#4 (Chronic Myelogenous Leukemia):ti,ab,kw OR (Leukemias, Ph1 Positive Myelogenous):ti,ab,kw OR (Myelogenous Leukemias, Chronic):ti,ab,kw OR (Chronic Granulocytic Leukemia):ti,ab,kw OR (Leukemia, Myelogenous, Ph1 Positive):ti,ab,kw (Word variations have been searched)<br>#5 (Leukemia, Philadelphia-Positive Myeloid):ti,ab,kw OR (Ph1 Positive Myeloid Leukemia):ti,ab,kw OR (Myeloid Leukemia, Chronic):ti,ab,kw OR (Leukemia, Myeloid, Ph1 Positive):ti,ab,kw OR (Leukemia, Granulocytic, Chronic):ti,ab,kw (Word variations have been searched)<br>#6 (Leukemia, Ph1 Positive Myeloid):ti,ab,kw OR (Chronic Myelocytic Leukemia):ti,ab,kw OR (Myeloid Leukemias, Ph1 Positive):ti,ab,kw OR (Granulocytic Leukemias, Chronic):ti,ab,kw OR (Myeloid Leukemia, Philadelphia Positive):ti,ab,kw (Word variations have been searched)<br>#7 #2 or #3 or #4 or #5 or #6<br>#8 #1 and #7	
	Nilotinib	#1 (nilotinib):ti,ab,kw OR (Tasigna):ti,ab,kw OR (AMN107):ti,ab,kw (Word variations have been searched)<br>#2 (Leukemia, Myelogenous, Chronic, BCR-ABL Positive):ti,ab,kw OR (Philadelphia-Positive Myeloid Leukemia):ti,ab,kw OR (Myeloid Leukemias, Chronic):ti,ab,kw OR (Leukemia, Myelogenous, Ph1 Positive):ti,ab,kw OR (Leukemias, Chronic Granulocytic):ti,ab,kw (Word variations have been searched)<br>#3 (Leukemia, Myelogenous, Chronic, BCR-ABL Positive):ti,ab,kw OR (Myelogenous Leukemias, Ph1 Positive):ti,ab,kw OR (Leukemia, Myeloid, Philadelphia Positive):ti,ab,kw OR (Myelocytic Leukemias, Chronic):ti,ab,kw OR (Granulocytic Leukemia, Chronic):ti,ab,kw (Word variations have been searched)	265

© 2021 Wang Z et al. *JAMA Network Open.*

		#4 (Chronic Myelogenous Leukemia):ti,ab,kw OR (Leukemias, Ph1 Positive Myelogenous):ti,ab,kw OR (Myelogenous Leukemias, Chronic):ti,ab,kw OR (Chronic Granulocytic Leukemia):ti,ab,kw OR (Leukemia, Myelogenous, Ph1 Positive):ti,ab,kw (Word variations have been searched)<br>#5 (Leukemia, Philadelphia-Positive Myeloid):ti,ab,kw OR (Ph1 Positive Myeloid Leukemia):ti,ab,kw OR (Myeloid Leukemia, Chronic):ti,ab,kw OR (Leukemia, Myeloid, Ph1 Positive):ti,ab,kw OR (Leukemia, Granulocytic, Chronic):ti,ab,kw (Word variations have been searched)<br>#6 (Leukemia, Ph1 Positive Myeloid):ti,ab,kw OR (Chronic Myelocytic Leukemia):ti,ab,kw OR (Myeloid Leukemias, Ph1 Positive):ti,ab,kw OR (Granulocytic Leukemias, Chronic):ti,ab,kw OR (Myeloid Leukemia, Philadelphia Positive):ti,ab,kw (Word variations have been searched)<br>#7 #2 or #3 or #4 or #5 or #6<br>#8 #1 and #7	
	Ponatinib	#1 (Ponatinib):ti,ab,kw OR (ponatinib hydrochloride):ti,ab,kw OR (AP24534):ti,ab,kw OR (AP-24534):ti,ab,kw OR (Iclusig):ti,ab,kw (Word variations have been searched)<br>#2 (Leukemia, Myelogenous, Chronic, BCR-ABL Positive):ti,ab,kw OR (Philadelphia-Positive Myeloid Leukemia):ti,ab,kw OR (Myeloid Leukemias, Chronic):ti,ab,kw OR (Leukemia, Myelogenous, Ph1 Positive):ti,ab,kw OR (Leukemias, Chronic Granulocytic):ti,ab,kw (Word variations have been searched)<br>#3 (Leukemia, Myelogenous, Chronic, BCR-ABL Positive):ti,ab,kw OR (Myelogenous Leukemias, Ph1 Positive):ti,ab,kw OR (Leukemia, Myeloid, Philadelphia Positive):ti,ab,kw OR (Myelocytic Leukemias, Chronic):ti,ab,kw OR (Granulocytic Leukemia, Chronic):ti,ab,kw (Word variations have been searched)<br>#4 (Chronic Myelogenous Leukemia):ti,ab,kw OR (Leukemias, Ph1 Positive Myelogenous):ti,ab,kw OR (Myelogenous Leukemias, Chronic):ti,ab,kw OR (Chronic Granulocytic Leukemia):ti,ab,kw OR (Leukemia, Myelogenous, Ph1 Positive):ti,ab,kw (Word variations have been searched)<br>#5 (Leukemia, Philadelphia-Positive Myeloid):ti,ab,kw OR (Ph1 Positive Myeloid Leukemia):ti,ab,kw OR (Myeloid Leukemia, Chronic):ti,ab,kw OR (Leukemia, Myeloid, Ph1 Positive):ti,ab,kw	39

© 2021 Wang Z et al. *JAMA Network Open.*

		OR (Leukemia, Granulocytic, Chronic):ti,ab,kw (Word variations have been searched)<br>#6 (Leukemia, Ph1 Positive Myeloid):ti,ab,kw OR (Chronic Myelocytic Leukemia):ti,ab,kw OR (Myeloid Leukemias, Ph1 Positive):ti,ab,kw OR (Granulocytic Leukemias, Chronic):ti,ab,kw OR (Myeloid Leukemia, Philadelphia Positive):ti,ab,kw (Word variations have been searched)<br>#7 #2 or #3 or #4 or #5 or #6<br>#8 #1 and #7	
	Imatinib	#1 (Imatinib Mesylate):ti,ab,kw OR (Gleevec):ti,ab,kw OR (Glivec):ti,ab,kw OR (Methanesulfonate, Imatinib):ti,ab,kw OR (Mesylate, Imatinib):ti,ab,kw (Word variations have been searched)<br>#2 (Imatinib Methanesulfonate):ti,ab,kw OR (ST 1571):ti,ab,kw OR (ST1571):ti,ab,kw OR (STI571):ti,ab,kw OR (STI-571):ti,ab,kw (Word variations have been searched)<br>#3 (STI 571):ti,ab,kw OR (CGP57148B):ti,ab,kw OR (CGP 57148):ti,ab,kw OR (CGP57148):ti,ab,kw OR (CGP-57148):ti,ab,kw (Word variations have been searched)<br>#4 #1 or #2 or #3<br>#5 (Leukemia, Myelogenous, Chronic, BCR-ABL Positive):ti,ab,kw OR (Philadelphia-Positive Myeloid Leukemia):ti,ab,kw OR (Myeloid Leukemias, Chronic):ti,ab,kw OR (Leukemia, Myelogenous, Ph1 Positive):ti,ab,kw OR (Leukemias, Chronic Granulocytic):ti,ab,kw (Word variations have been searched)<br>#6 (Leukemia, Myelogenous, Chronic, BCR-ABL Positive):ti,ab,kw OR (Myelogenous Leukemias, Ph1 Positive):ti,ab,kw OR (Leukemia, Myeloid, Philadelphia Positive):ti,ab,kw OR (Myelocytic Leukemias, Chronic):ti,ab,kw OR (Granulocytic Leukemia, Chronic):ti,ab,kw (Word variations have been searched)<br>#7 (Chronic Myelogenous Leukemia):ti,ab,kw OR (Leukemias, Ph1 Positive Myelogenous):ti,ab,kw OR (Myelogenous Leukemias, Chronic):ti,ab,kw OR (Chronic Granulocytic Leukemia):ti,ab,kw OR (Leukemia, Myelogenous, Ph1 Positive):ti,ab,kw (Word variations have been searched)<br>#8 (Leukemia, Philadelphia-Positive Myeloid):ti,ab,kw OR (Ph1 Positive Myeloid Leukemia):ti,ab,kw OR (Myeloid Leukemia, Chronic):ti,ab,kw OR (Leukemia, Myeloid, Ph1 Positive):ti,ab,kw OR (Leukemia, Granulocytic, Chronic):ti,ab,kw (Word variations have been searched)	329

© 2021 Wang Z et al. *JAMA Network Open.*

		#9 (Leukemia, Ph1 Positive Myeloid):ti,ab,kw OR (Chronic Myelocytic Leukemia):ti,ab,kw OR (Myeloid Leukemias, Ph1 Positive):ti,ab,kw OR (Granulocytic Leukemias, Chronic):ti,ab,kw OR (Myeloid Leukemia, Philadelphia Positive):ti,ab,kw (Word variations have been searched)<br>#10 #5 or #6 or #7 or #8 or #9<br>#11 #4 and #10	

© 2021 Wang Z et al. *JAMA Network Open.*

eTable 2: Characteristics of included studies and quality assessment.

Authors (year)	Trial register	Trial desig	Country	Treatment arm	Number of	Population	Median age	Male,n (%)	Jadad
Gambacorti-Passerini et al,[23]	NCT00574873	Phase III; RCT	30 countries	bosutinib	250	chronic phase CML	48(19-	149(59.	2
				imatinib 400	252		47(18-89)	135(53.6)	
Cortes et al,[24] 2018	NCT02130557	Phase III;	27 countri	bosutinib	268	chronic phase	53(18-	156(58.	2
				imatinib	268		53(19-	155(57.	
Hjorth-Hansen et al,[25] 2014	NCT00852566	Phase II; RCT	Finland, Norway	dasatinib	22	chronic phase CML	53(29-	7(31.8)	2
				imatinib 400	24		58(38-78)	15(62.5)	
Kantarjian et al,[26]	NCT00481247	Phase III;	28 countri	dasatinib	259	chronic phase	46(18-	144(56)	3
				imatinib	260		49(18-	163(63)	
Radich et al,[27] 2012	NCT00070499	Phase II;	Canada, United	dasatinib	123	chronic phase	47(18-	75(61)	3
				imatinib	123		50(19-	72(58.5)	
Hughes et al,[28] 2014	NCT00760877	Phase III;	6 countri	nilotinib	104	chronic phase	46 (23-	71(68.3)	2
				imatinib	103		52 (19-	65(63.1)	
Saglio et al,[29] 2010	NCT00471497	Phase III;	36 countri	nilotinib	563	chronic phase	47(18-	333(59.	3
				imatinib	283		46(18-	158(56)	
Wang et al,[30] 2015	NCT01275196	Phase III;	China	nilotinib	134	chronic phase	41(18-	91(67.9)	3
				imatinib	133		39(19-	81(60.9)	
Lipton et al,[31] 2016	NCT01650805	Phase III;	25 countri	ponatinib	154	chronic phase	55 (18-	97(63)	3
				imatinib	152		52 (18-	92(61)	

Abbreviation: CML = Chronic myeloid leukemia.

© 2021 Wang Z et al. *JAMA Network Open.*

eFigure 1: Flow diagram of study selection according to PRISMA statement.

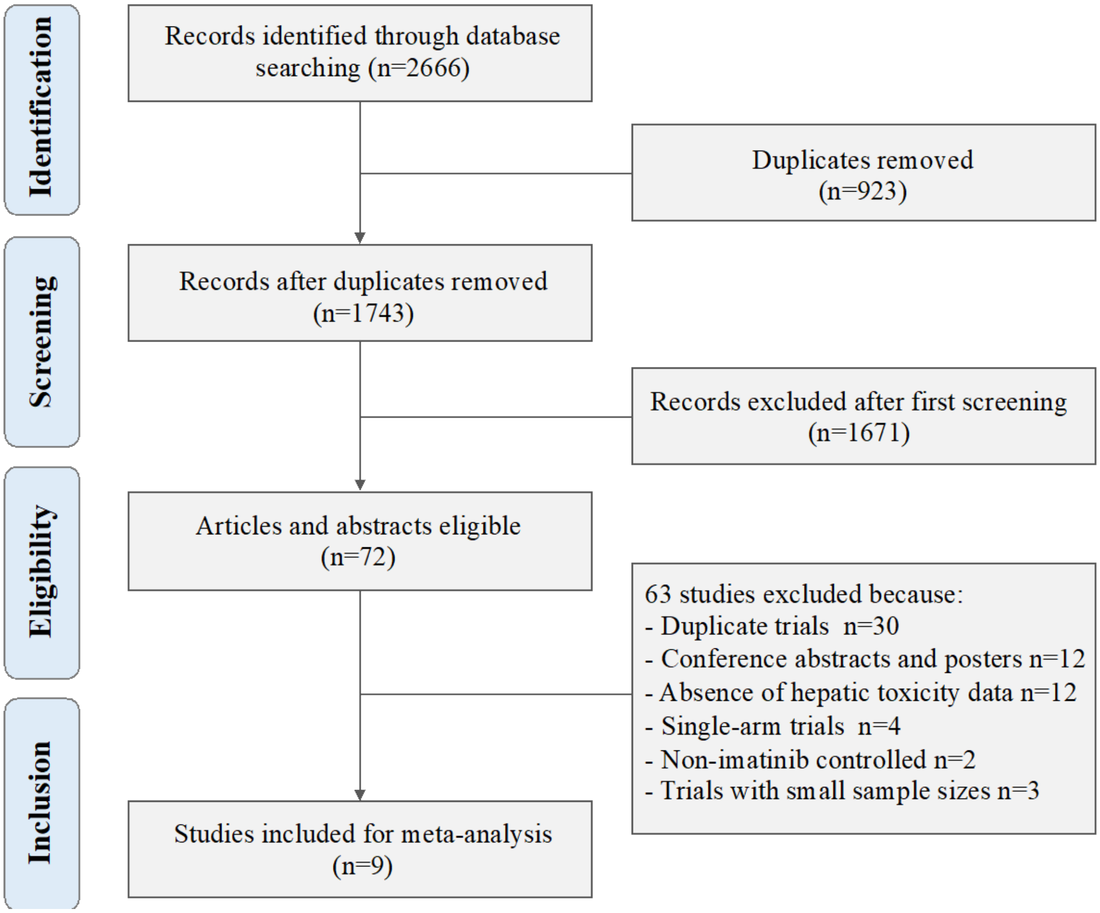

© 2021 Wang Z et al. *JAMA Network Open.*

eFigure 2: Evaluation of publications bias.

A. All grades alanine aminotransferase (ALT) elevation

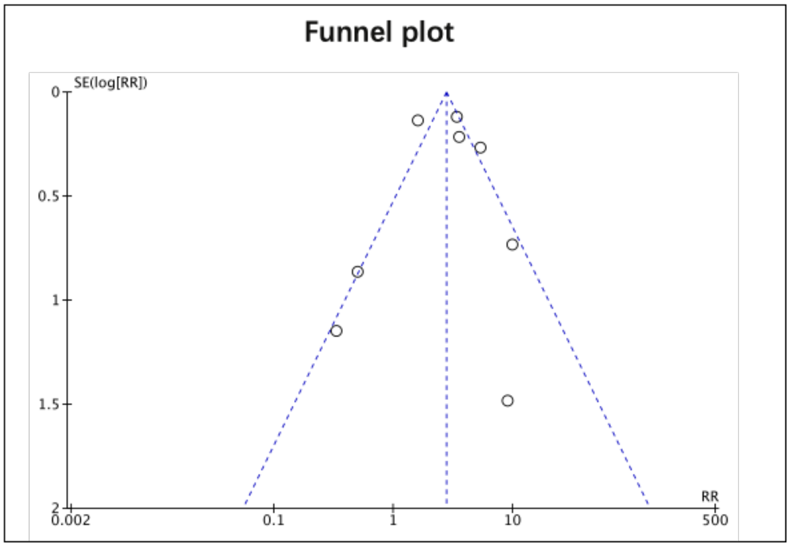

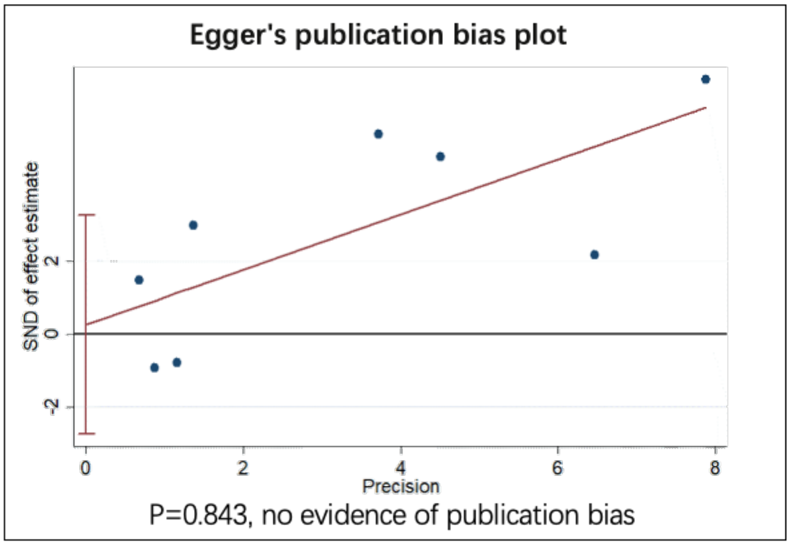

© 2021 Wang Z et al. *JAMA Network Open.*

B. Grade 3-4 ALT elevation

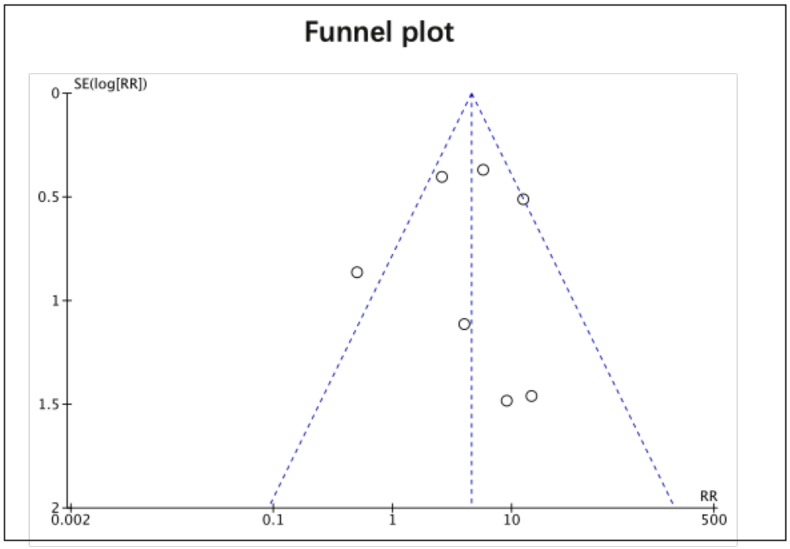

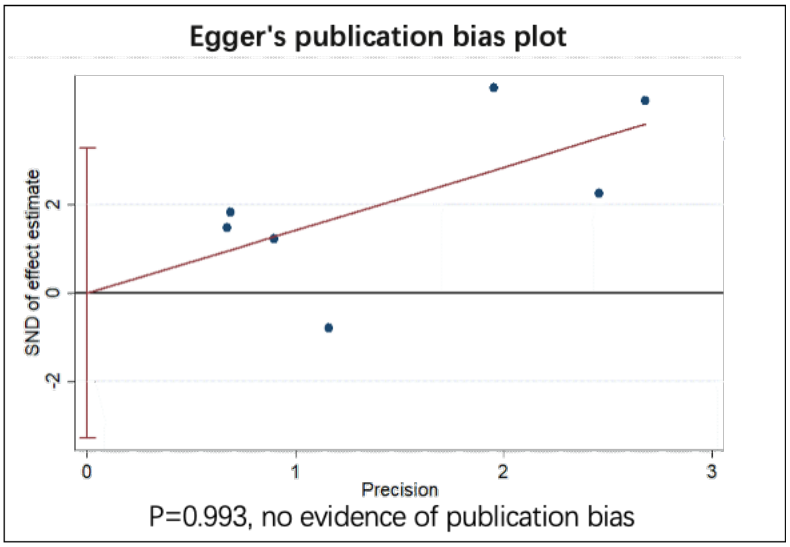

© 2021 Wang Z et al. *JAMA Network Open.*

C. All grades AST elevation

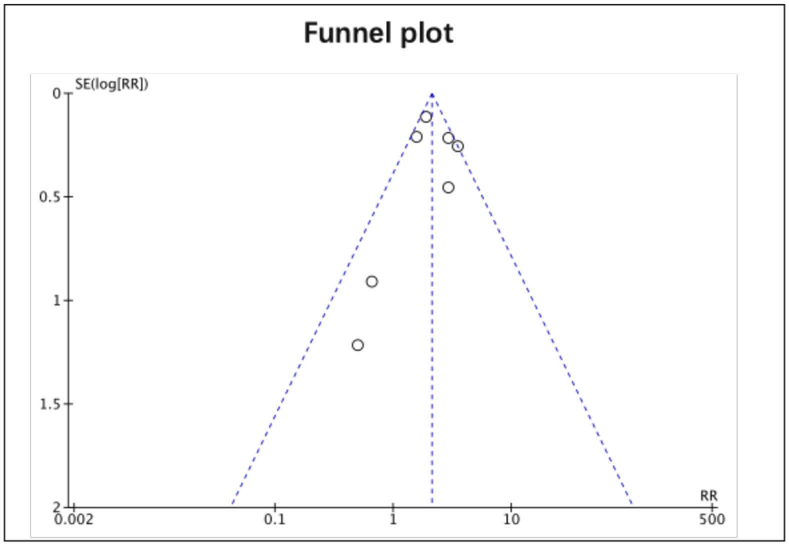

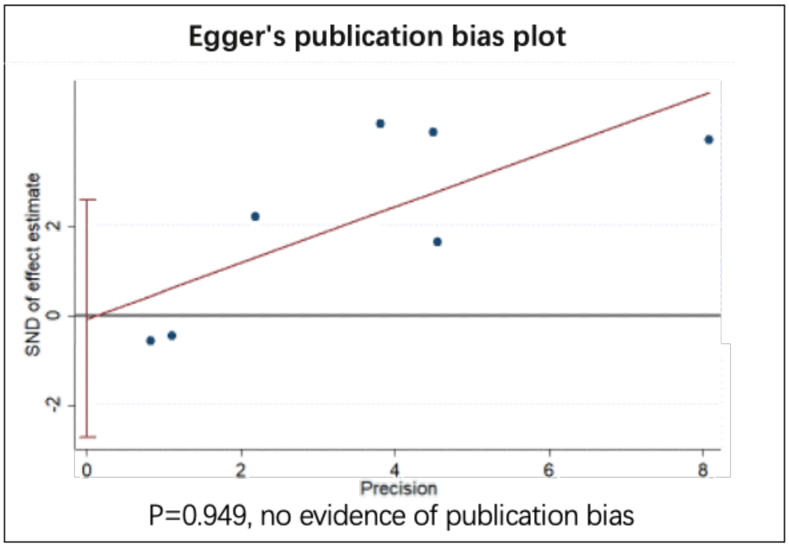

© 2021 Wang Z et al. *JAMA Network Open.*

D. Grade 3-4 aspartate aminotransferase (AST) elevation

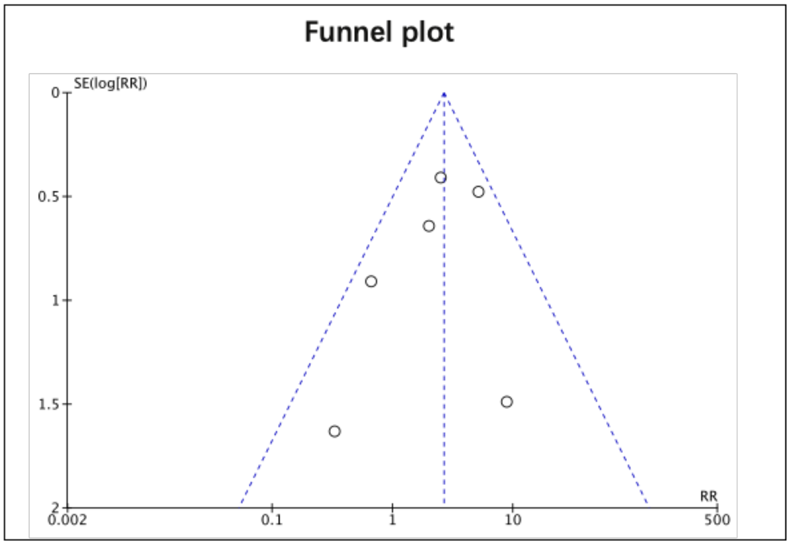

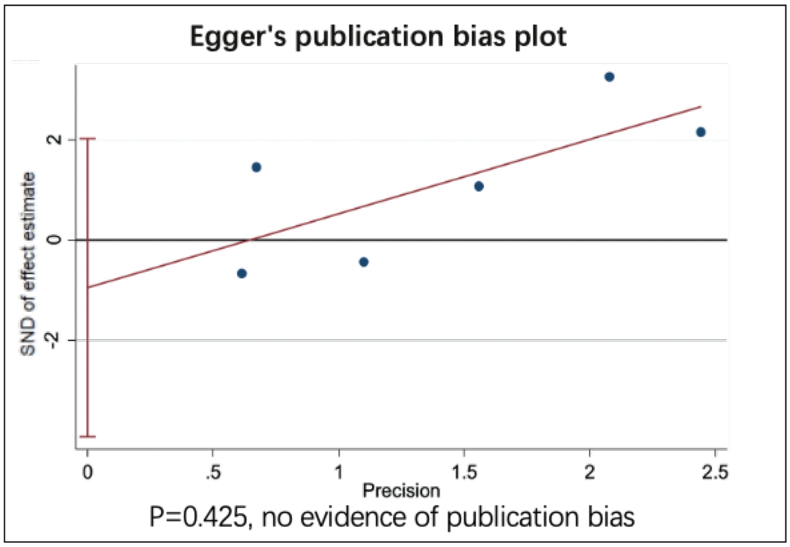

© 2021 Wang Z et al. *JAMA Network Open.*

E. Overall survival (OS)

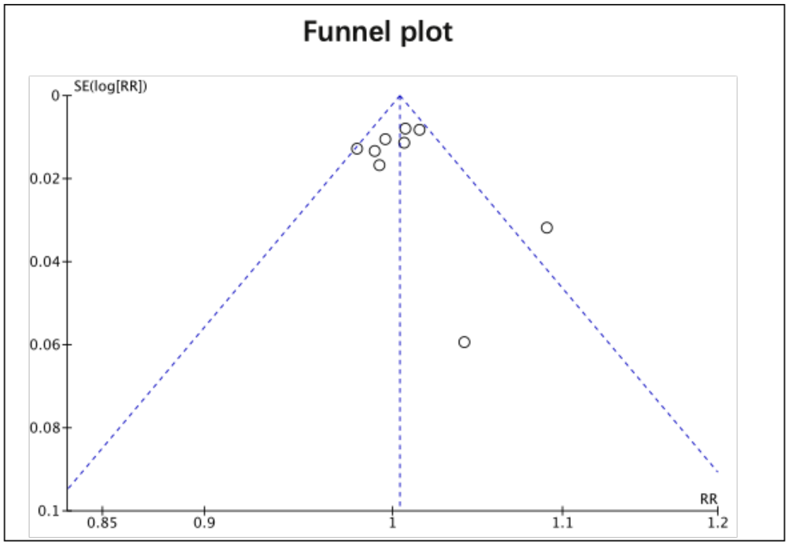

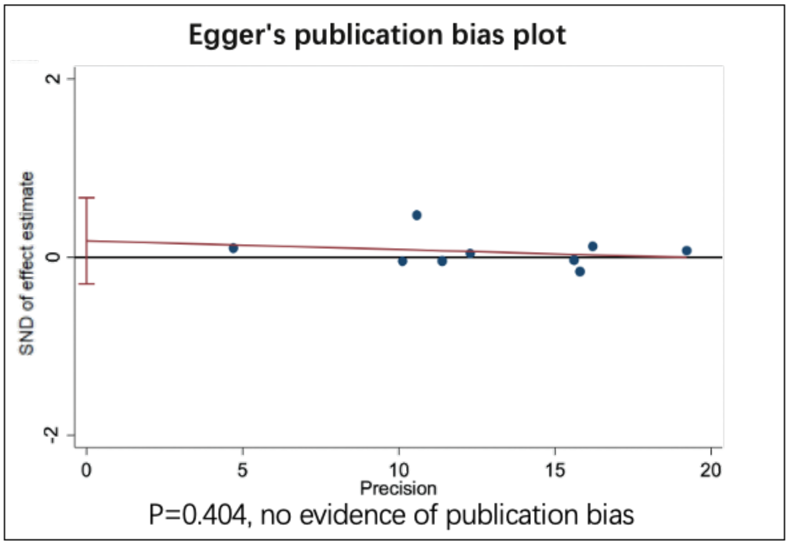

© 2021 Wang Z et al. *JAMA Network Open.*

F. Major molecular response (MMR)

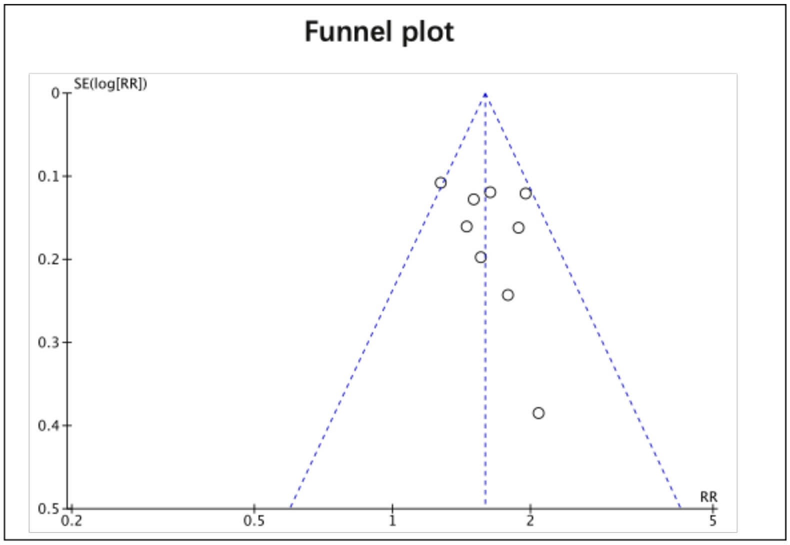

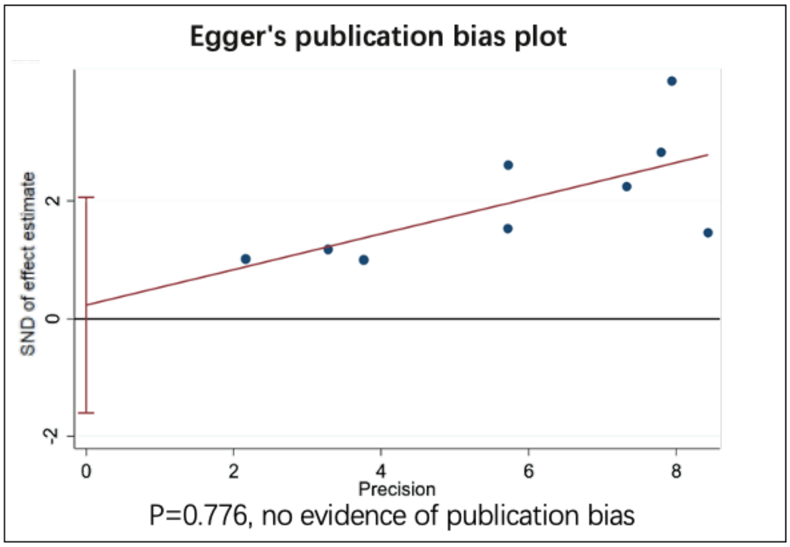

© 2021 Wang Z et al. *JAMA Network Open.*
